# Hyposalivation but not Sjögren’s syndrome associated with microbial dysbiosis in women

**DOI:** 10.3389/fmicb.2023.1240891

**Published:** 2023-10-06

**Authors:** Carlos Saúco, Maria J. Rus, María R. Nieto, Carolina Barros, Cristiane Cantiga-Silva, Débora Lendines-Cordero, Marta Calderer-Ortiz, Miriam Zurita-García, Santiago Arias-Herrera, Loreto Monsalve-Guil, Juan José Segura-Egea, Aurea Simon-Soro

**Affiliations:** ^1^Department of Stomatology, Faculty of Dentistry, University of Seville, Seville, Spain; ^2^Department of Preventive and Restorative Dentistry, Dental School, São Paulo State University (UNESP), Araçatuba, Brazil; ^3^Instituto Para el Estudio de la Biología de la Reproducción Humana (INEBIR), Seville, Spain; ^4^Department of Dentistry, Faculty of Health Sciences, Universidad Europea de Valencia, Valencia, Spain

**Keywords:** hyposalivation, Sjögren’s syndrome, aging, saliva, estrogen, *Prevotella*, women’s health

## Abstract

**Background:**

Saliva modulates the environment of the oral biofilm through pH buffer, microbial attachment to host surfaces, and nutritional source. The ecology of stress occurs when a physical factor adversely impacts an ecosystem or its biotic components. Therefore, reduced salivary flow can affect oral-host balance. The leading causes of hyposalivation include disease-associated Sjögren’s syndrome (SS) and menopausal women as aging-associated. However, little is known about the oral microbiome integrated with sex hormones in hyposalivation. This study aimed to characterize the hyposalivation microbiome caused by aging or disease affecting the salivary glands in women.

**Methods:**

We included 50 women older than 40 years of age in any menopausal phase. We collected stimulated saliva from 25 women diagnosed with SS (SS) and 25 without SS (non-SS). The bacterial profile of the patients was obtained by 16S rRNA sequencing. Bioinformatics analysis used machine learning to analyze the cohort’s signs, symptoms, and bacterial profile. Salivary estradiol as a sex hormone variation level was determined.

**Results:**

We obtained that 79% of the SS group, and 52% of the non-SS group had hyposalivation. We found a negatively correlated *Prevotella*-age and *Rothia*-estradiol in the SS group. Highlight, we found that the cause of the hyposalivation in the study did not explain differences in microbial diversity comparing non-SS and SS groups. Therefore, microbial communities found in hyposalivation but not related to systemic conditions suggest that changes in the oral environment might underpin host-microbial balance.

**Conclusion:**

The salivary microbiome was similar in women with and without SS. However, hyposalivation showed two distinctive clusters associated with the bacterial population profiles. Our study suggests that local ecological disturbances could drive the change in the microbiome.

## 1. Introduction

Hyposalivation is the condition characterized by a reduced production of saliva, which can be caused by several disorders (Smith et al., 2007; Einhorn et al., 2020). Hyposalivation has an overall prevalence of approximately 22%, with a higher incidence in women than in men and increases with age (Agostini et al., 2018; Pina et al., 2020; Pérez-Jardón et al., 2022). Reduced saliva production can be caused by various disorders, including pathological or age-related factors, as reported in previous studies (Ogle, 2020; Pina et al., 2020). Pathological hyposalivation can be triggered by autoimmune diseases such as Sjögren’s syndrome or Mikulicz Syndrome, as well as by Parkinson’s disease and the formation of neoplasms or bacterial infections of the salivary glands, leading to obstruction (sialadenitis) (Ogle, 2020). Age-related hyposalivation is linked to polymedication, with different drugs found to be associated with salivary gland dysfunction (Einhorn et al., 2020; Pina et al., 2020). Aging patients often report subjective symptoms of this condition, known as xerostomia (Smith et al., 2007; Pérez-Jardón et al., 2022). Saliva has many functions in the oral cavity that include moistening, lubrication, and protection of the oral cavity and esophagus, digestion, tasting, and smelling. It also plays a crucial role in maintaining oral health due to the presence of antimicrobial substances that maintain oral microbiome homeostasis (Holmberg and Hoffman, 2014; Dawes et al., 2015; Marsh et al., 2016). Therefore, salivary flow reduction can also affect microbial colonization, which ultimately contributes to biofilm formation (Marsh et al., 2016).

The oral cavity contains a diverse microbial community, consisting of more than 750 identified bacterial species (Human Oral Microbiome Database, n.d.). This community undergoes compositional changes over time, with the most distinct differences seen between childhood and late adulthood, and a stable period in between (Willis et al., 2022). A lower diversity of microbiota was found in elderly people and several taxa had been associated with age (Schwartz et al., 2021; Wells et al., 2022). A disruption in the balance of the oral microbial community can lead to dysbiosis, whether caused by an overgrowth of microorganisms or changes in the local host response. The dysbiotic state creates an environment that facilitates the development of disease (Roberts and Darveau, 2015). Notably, oral dysbiosis has been linked not only to oral diseases, such as caries and periodontal diseases but also to systemic health issues (Peng et al., 2022). For instance, the oral microbiome has been linked to rheumatoid arthritis and pulmonary diseases such as asthma, lung cancer, or pneumonia (Pathak et al., 2021; Sedghi et al., 2021).

Several factors can affect microbial populations in the oral cavity, including disease status, menstrual status, and salivary flow rate. For example, hyposalivation-affected individuals have been found to have an altered oral microbiome compared to those with normal salivary flow (Rusthen et al., 2019). Studies have also shown that SS can affect microbiome composition, with changes observed in the relative abundance of certain bacterial groups (Rusthen et al., 2019; Singh et al., 2021). However, the specific effects of SS on the oral microbiome are not yet fully understood, as conflicting results have been reported in the literature (Rusthen et al., 2019; Sharma et al., 2020; Singh et al., 2021). Menstrual status is a well-known factor that can cause significant changes in the vaginal microbiota (Farage et al., 2010). Studies have shown that it can also affect the oral microbiota by altering its richness and diversity during different menstrual phases (Krog et al., 2022). These changes are largely driven by estrogen, a hormone that is known to play a key role in the menstrual cycle. This hormone has been linked to the capacity of bacteria to coaggregate and participate in biofilm formation (Fteita et al., 2014).

In this study, we investigated the relationship between oral microbiota and hyposalivation in aging women considering different conditions such as menstrual state, hyposalivation, and SS. We collected clinical data and oral samples from these patients to analyze the bacteriome of their oral microbiota using 16S rRNA sequencing. The goal of this investigation was to identify potential microbial markers of these conditions and to elucidate the mechanisms linking oral microbiota dysbiosis with salivary gland hypofunction. This study could provide information on the role of the oral microbiota in systemic health and help identify new targets for the diagnosis and treatment of oral health related to women.

## 2. Materials and methods

### 2.1. Study design and sample selection

This is a case–control study conducted in the Dental Schools of the University of Seville and the European University of Valencia from January 2020 to June 2021. Saliva samples and clinical data were collected from a cohort of 25 women with Sjögren’s syndrome and 25 controls of the same age and sex. The inclusion criteria were women between 40 and 70 years of age diagnosed with Sjögren syndrome according to the AEGG and ACR-EULAR 2016 guidelines. Exclusion criteria included incapacitating disease for independent collection of clinical data or biological samples, age less than 40 or over 70 years, antibiotics or antifungals in the last 3 months before sample collection, or mouthwashes with oral antiseptics in the month before sampling. Men and women who, according to the inclusion criteria, did not sign the informed consent for the study were also excluded. Subject recruitment was carried out between patients at the dental clinic of the Faculty of Dentistry of the University of Seville and the European University of Valencia.

### 2.2. Sample collection

Two saliva samples per patient were collected to determine pH, salivary flow rate, and estradiol levels, and to sequence the oral microbiome. First, saliva by drooling (SA), in a 50 mL sterile tube for 5 min. Subsequently, saliva was stimulated saliva (ST) by chewing paraffin pellets, in another 50 mL sterile tube for 5 min (Alvariño et al., 2021). The samples were kept at −80° C until processing.

### 2.3. Salivary measurement assays

The salivary flow was measured by decantation in a calibrated test tube for SA and ST. A normal salivary flow rate was established at 0.3 mL/min for SA and 1.5 mL/min for ST (Bartold et al., 2017). The salivary pH was determined in ST with a pH meter (Crison Basic 20). Estrogen levels were obtained using ST sample using an enzyme immunoassay kit for measuring salivary 17-β estradiol following the manufacturer’s recommended protocol (Salimetrics. State College, PA). The assays were performed in duplicate with the average of the duplicate results used in the analyzes. Salivary estradiol values were measured in picograms per milliliter. Several samples were not measured for the estradiol assay due to the limited volume of saliva in subjects with extreme hyposalivation (7 samples). The other 3 samples were measured but obtained values below the detection limit. The normal levels of salivary estradiol ranged from 8–10 pg./mL based on the literature (Gavrilova and Lindau, 2009; Kozloski et al., 2014).

### 2.4. Collecting clinical data

To ensure consistency and standardization of sampling and oral clinical data collection, participating dentists underwent extensive calibration training sessions. These sessions were designed to align their sampling techniques, methodologies, and clinical criteria, thereby guaranteeing uniformity in the data collection process. Cohen’s Kappa statistics were obtained for the degree of concordance between examiners (k = 0 0.83–0.71).

Oral and dental clinical data collection was based on oral mucosal examination and assessment of dental and periodontal status using a dental examination kit, dental mirror, caries probe, and millimeter periodontal probe. First, the alterations observed in oral structures covered by mucosa were recorded, specifying their location. Next, using the dental mirror and caries probe, the dental status was evaluated according to the DMFT index (Fejerskov and Kidd, 2008). This involved documenting the presence of caries lesions, fillings, tooth loss, fissures, fractures, hypoplasias, abutment teeth, dental crowns, and unerupted teeth. Periodontal status was determined through the Basic Periodontal Exam which provides a basis for a simple and fast examination and allows the evaluation of treatment needs (Dietrich et al., 2019). For data collection, the dentition is divided into canine-limited sextants, without recording the third molars. Sextants with only one tooth are excluded and the tooth is added to the adjacent sextant. The millimetered periodontal probe is introduced between the tooth and the gingiva to determine the depth of the gingivodental sulcus relative to the level of the gingival margin. Six probing points are performed on each tooth: mesial, midpoint, and distal of the vestibular and palatal/lingual side. The presence of dental calculus or other plaque retention factors is also assessed.

For coding purposes in the Basic Periodontal Exam, code 0 is assigned to sextants that do not have pockets of 4 mm or deeper, there is no calculus, overhanging fillings, and no bleeding after probing. Code 1 is for the sextants that, under these healthy conditions, show bleeding after probing. Code 2 is assigned to the sextants where dental calculus or other plaque retention factors such as overhanging fillings are observed. Code 3 is given to the sextant where the maximum probing depth in one or more teeth is between 4–6 mm. Code 4 is when one or more teeth have a probing depth greater than 6 mm. The * code is given to the sextant where there is an attachment loss of 7 mm or more, or if furcation involvement exists. Each sextant is assigned the highest value obtained.

Based on this scoring, the need for treatment is determined. The management of sextants with codes 0, 1, and 2 is as follows: Code 0, no treatment is required. Code 1 can be managed through oral hygiene instructions and supragingival prophylaxis. For Code 2, oral hygiene instructions, supragingival and selected subgingival prophylaxis, and removal of overhanging fillings are needed. In the presence of code 3, 4, or *, a thorough periodontal examination (periodontogram, plaque index, gingival index, tooth mobility, and panoramic radiography) is required for further information. Clinical management will consist of full-mouth prophylaxis and scaling and root planing for sextants with code 3. Patients with sextants scored as Code 4 or * will need comprehensive supragingival and subgingival prophylaxis, scaling and root planing, and periodontal surgery, with a continued emphasis on plaque control.

The basic health questionnaire was designed to collect the medical and pharmacological history of the participants and other aspects of their lifestyle, exercise, diet, toxic habits (tobacco and alcohol), and coffee. A menopause questionnaire was also included to determine the stage of menopause and the use of hormone therapy after the international classification of menopausal stages, premenopause (periodical menses), perimenopause (no periodical menses for the last 12 months), postmenopause (menses cessation >12 months) (The North American Menopause Society, 2023).

### 2.5. DNA extraction and 16S amplification

Microbial DNA was extracted from samples using the DNeasy Powersoil Pro Kit (Qiagen) following the manufacturer’s instructions. The input gDNA 2 ng was amplified by PCR with 5x reaction buffer, 1 mM of dNTP mix, 500 nM each of the universal F/R PCR primer, and Herculase II fusion DNA polymerase (Agilent Technologies, Santa Clara, CA). The cycle condition for the 1st PCR was 3 min at 95 ° C for heat activation, and 25 cycles of 30 s at 95°C, 30 s at 55 ° C, and 30 s at 72°C, followed by a final extension of 5 min at 72 ° C. Sequencing libraries were prepared by amplifying the V3-V4 region of the 16S rRNA gene using the universal F/R PCR primer (Fw: 5’-TCGTCGGCAGCGTCAGATGTGTATAAGAGACAGCCTACGGGNGGCWGCAG; Rv: 5’-GTCTCGTGGGCTCGGAGATGTGTATAAGAGACAGGACTACHVGGGTATCTAATCC). The amplicons were purified with AMPure beads (Agencourt Bioscience, Beverly, MA). The purified product was quantified using qPCR according to the qPCR Quantification Protocol Guide (KAPA Library Quantification kits for Illumina Sequencing platforms) and qualified using TapeStation D1000 ScreenTape (Agilent Technologies, Waldbronn, Germany). Sequencing was performed using the MiSeqTM platform (Illumina, San Diego, United States). We included 3 samples as negative controls to test the contamination from DNA extraction reagents. Also, we sequenced a positive control using ZymoBIOMICS Microbial Community DNA Standard II (ZYMO) to test the amplification and microbial analysis procedure.

### 2.6. Bacteriome analysis

Microbiome analysis was performed with the bioinformatics platform QIIME2 v.2022.8.3 (Bolyen et al., 2019). Raw 16S rRNA gene sequences were quality filtered, denoised, and dereplicated with DADA2 using the denoise-paired plugin, designed for paired-end demultiplexed sequences (Callahan et al., 2016). Due to a decrease in quality, the forward and reverse read sequences were truncated at positions 278 and 205, respectively. The first 21 bases of the 5′ end of the sequences were also trimmed because of the low quality. Sequences with a Phred quality score below 20 were excluded from downstream analyzes. The amplified sequence variants (ASVs) obtained were aligned with the Greengenes 13_8 99% OTU reference sequence database using the classify sklearn Naive Bayes taxonomy classifier (McDonald et al., 2012; Bokulich et al., 2018). Alpha and Beta diversity was explored from a phylogenetic tree constructed with the Fasttree method based on the alignment of ASVs obtained with Mafft (Price et al., 2010; Katoh and Standley, 2013). Diversity metrics (Faith’s phylogenetic diversity, UniFrac distance, Jaccard distance, and Bray–Curtis dissimilarity) were estimated using a diversity plugin at a sampling depth of 1,670 sequences per sample. Statistical analysis was carried out using R v4.2.2 (R Foundation for Statistical Computing, Austria, 2022). To assess the association between microbial and clinical variables, library rstatix v0.7.2 was used. Ggpubr v0.6.0 was used for the correlation coefficient using Pearson and the significance level. For alpha diversity, pairwise Wilcoxon rank sum between disease groups and salivary flow in Richness and Shannon indexes were considered. To analyze the differences between the disease and salivary flow groups in Beta diversity, a Permutational Multivariate Analysis of Variance (Anderson, 2017) was used. Library cluster v2.1.4 was installed to analyze the partition around medoids (Kaufman and Rousseuw, 1990).

## 3. Results

### 3.1. Hyposalivation in women over 40 years of age

**Figure 1:**
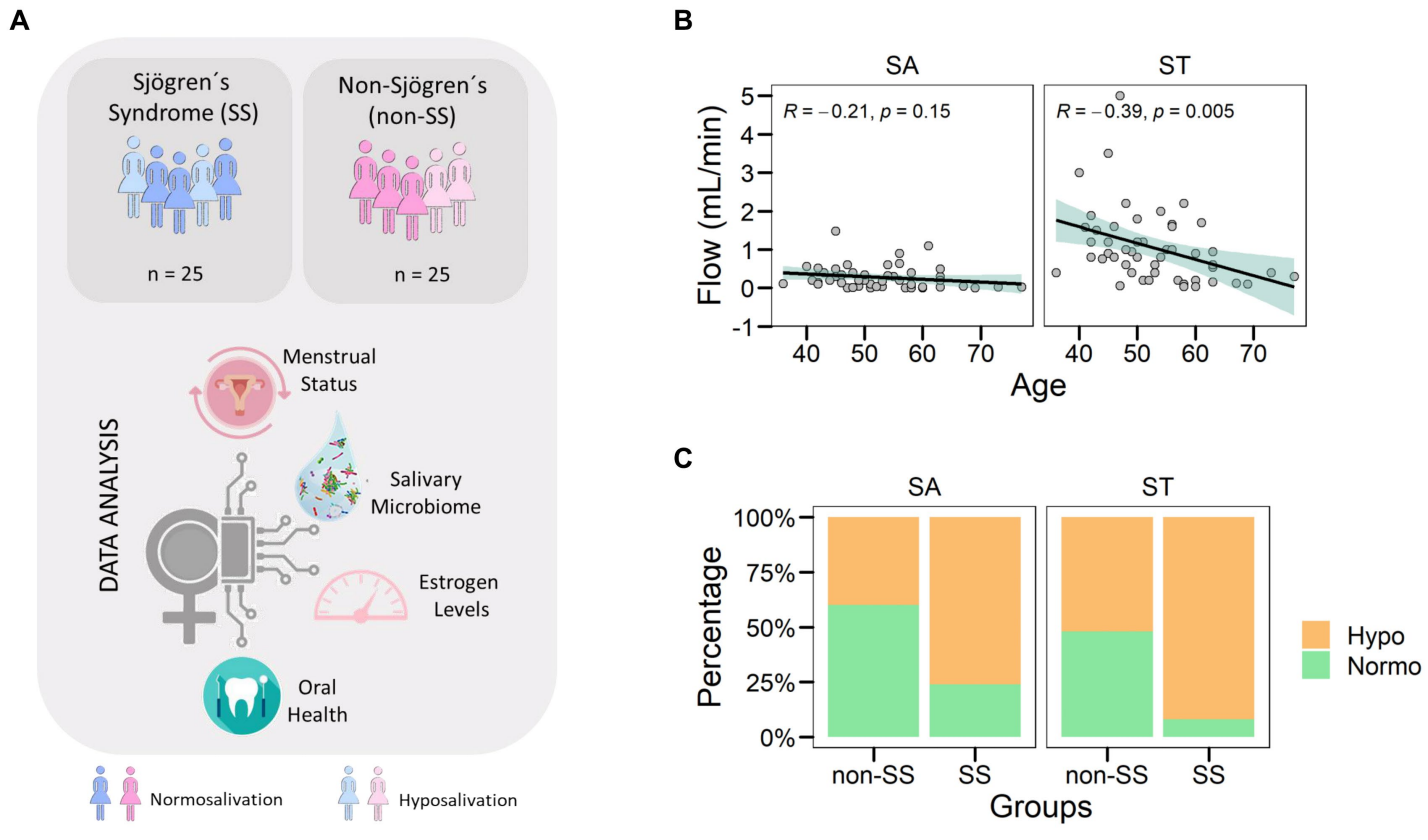
Hyposalivation in women over 40 years old. **(A)** Diagram showing the study workflow. Saliva samples were taken from a total of 50 women, 25 of them diagnosed with SS. After DNA extraction and sequencing, the microbiome population was obtained. Data were analyzed considering clinical data, disease condition, and age. **(B)** The correlation between age and salivary flow was assessed for both types of saliva collected. Each point corresponds to a sample. The green area corresponded to the 95% confidence interval of the linear model. **(C)** Distribution of samples by salivary types (SA and ST) and disease groups (non-SS and SS). The colors corresponded to salivary flow categories such as hyposalivation (Hypo) in yellow and normosalivation (Normo) in green.

To understand the relationship between hyposalivation and the oral microbiome in women, we collected saliva samples from 50 women over 40 years old. Of the total of 50 women, half were diagnosed with the known autoimmune disease Sjogren’s syndrome affecting the salivary glands (Figure 1A). We also collected medical data, oral clinical exam, salivary estradiol, and pH that allowed us to correlate the microbial profile with the oral ecological aspects of the oral fluid (Table 1).

**Table 1:** Characteristics of the study population.

Variable	Women	Women SS	*p*
Age (mean (SD))	51.32 (6.21)	54.44 (10.74)	0.216
Estradiol (mean (SD))	0.98 (0.90)	0.77 (1.00)	0.482
SA (mean (SD))	0.40 (0.34)	0.14 (0.18)	0.002**
ST (mean (SD))	1.46 (1.14)	0.63 (0.47)	0.002**
*Menstrual status*			
Premenopause	11 (44)	11 (44)	
Postmenopause	14 (56)	14 (56)	

Median, standard deviation and value of p of age, estrogen levels, SA, and ST. The distribution of patients with respect to their menstrual status is also included. (%) unless otherwise noted. SA, non-stimulated saliva; ST, stimulated saliva; SD, standard deviation.

First, we analyzed the salivary flow of the women included in this study to measure SA and ST. When we associated saliva types with age, we obtained a negative correlation of ST flow and age but not in SA flow suggesting that saliva production could be affected after stimuli (Figure 1B, *R* = −0.39, *p* = 0.005). Focusing on disease groups, we found an uneven distribution of reduced saliva flow rate (Hypo) in both saliva collected types (Figure 1C). In the non-SS group, the study population was distributed 43% in SA and 53% in ST, while SS had 76% in SA and 92% in ST had reduced salivary flow rate (Hypo). Therefore, SS had accumulative factors of disease and aging that could affect salivary gland tissue and function, resulting in the reduction of SA and ST.

### 3.2. Saliva microbiome in aging and disease

**Figure 2:**
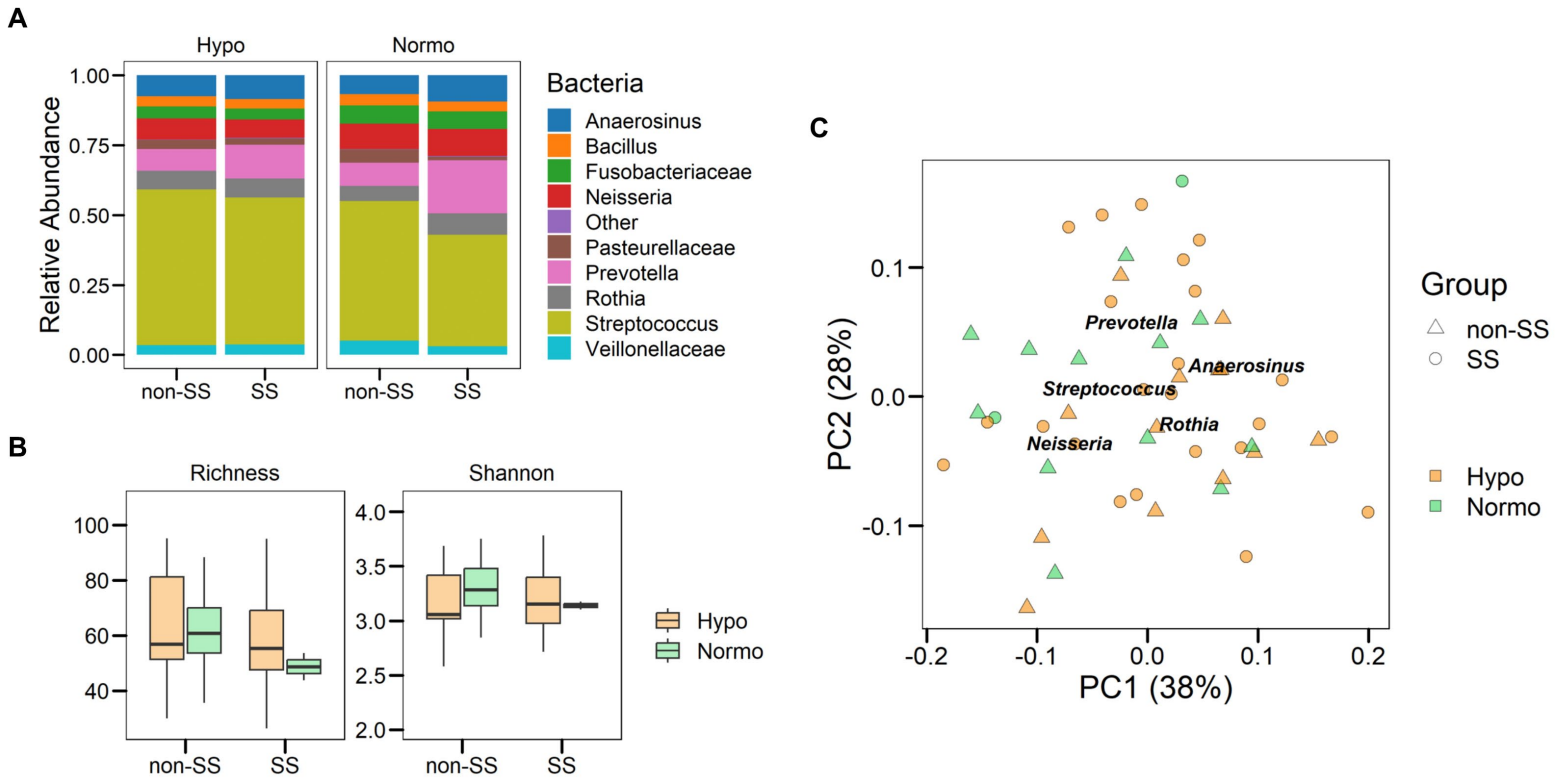
Saliva microbiome in aging and disease. **(A)** Relative abundance of bacterial taxa in Hypo and Normo as saliva flow categories considering SS and non-SS groups. Comparison of the abundances of the ten predominant bacteria detected after microbiome analysis with the relative abundance shown as a ratio. **(B)** Boxplot showing the alpha diversity richness and Shannon index. **(C)** Principal coordinate analysis showing the bacterial distribution according to health status and salivary condition. PC1 and PC2 explained 39 and 27% of the variance observed in the oral microbiome, respectively.

Hyposalivation not only reduces saliva volume but also might affect the biotic components of the oral cavity as its microbial community. To determine the microbiome in hyposalivation, we analyzed the stimulated saliva bacterial profile of the women included in the study. The microbial composition in hyposalivation (Hypo) and normosalivation (Normo) showed that the oral genera *Prevotella*, *Neisseria*, and *Rothia* highlighting *Streptococcus* are the most abundant taxa with 44% as the mean. When we characterized the bacteriome that separates women with SS, we found 16 and 10% for *Prevotella* for Hypos and Normo, respectively. However, the abundance of *Prevotella* decreased in the non-SS group by up to 8 and 7% for Hypo and Normo, respectively (Figure 2A). Then, our data showed a higher abundance of *Prevotella* related to SS condition.

Next, we analyze the alpha diversity of saliva flow and disease groups that showed no significant differences in richness or diversity for any of the groups (Figure 2B, *p* > 0.05). To assess whether the bacterial community was defined by saliva flow or disease in women, we analyzed beta diversity using weighted Unifrac distances (Figure 2C). We found a microbial community associated with saliva flow, while the diagnosis of Sjögren did not show a significant distribution (*p* = 0.001 and *p* = 0.35, respectively, PERMANOVA). Therefore, our results showed that the salivary bacterial community was independent of Sjögren’s syndrome. However, *Prevotella* and *Streptococcus* might be associated with a disease.

### 3.3. *Streptococcus* and *Prevotella* defined microbial clusters

**Figure 3:**
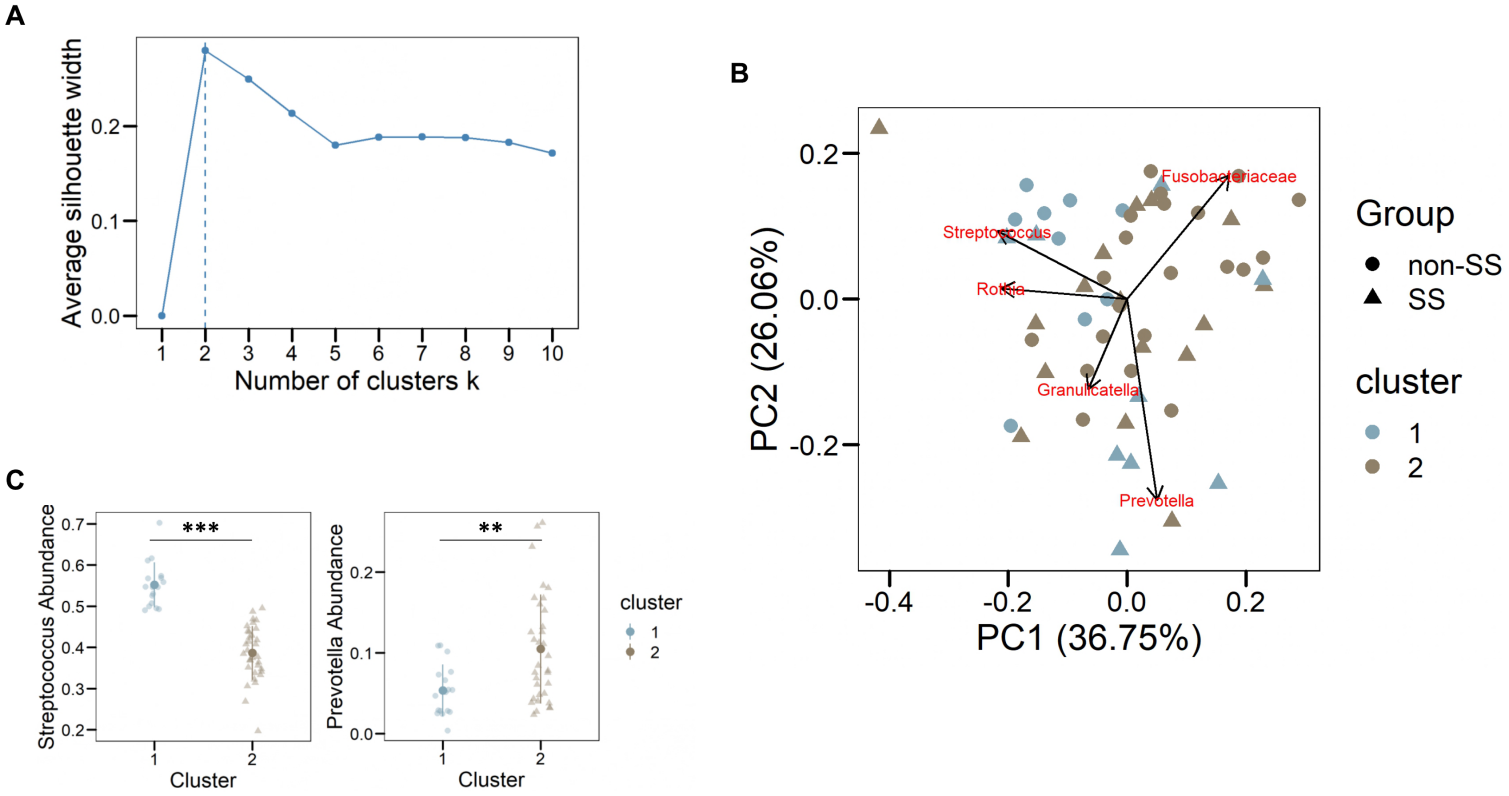
*Streptococcus* and *Prevotella* defined microbial clusters. **(A)** Number of clusters corresponding to the distribution of the oral microbiome according to PAM. The highest average silhouette width is found for two clusters, meaning that the best aggrupation is found for two clusters. **(B)** Principal component analysis with the distribution of the main bacterial taxa. The colors corresponded to the clusters assigned for each sample **(C)** Cluster distribution for the genera *Streptococcus* and *Prevotella* using the abundance of bacteria. ^**^ <0.01, ^***^ <0.001.

To assess the microbial profile of our cohorts independently of specific clinical features, we used an unsupervised classification method partition around medoids (Shi et al., 2022). The best number of k-medoids or clusters found in our data was k = 2 (Figure 3A). We obtained the main microorganisms separating the main 2 dimensions using Principal Component Analysis (Figure 3B). As a result, we obtained the main taxa separating the dimensions as *Streptococcus*, *Rothia*, *Granullicatella*, *Prevotella*, and *Fusobacteriaceae*. The bacterial genus Streptococcus (cursive) was associated with cluster 1 (Figure 3C left panel, *p* < 0.001) while *Prevotella* was associated with group 2 (Figure 3C right panel, *p* < 0.01). However, other taxa did not show significant differences in the cluster distribution. In particular, the non-SS group had a higher proportion of Streptococcus, while the SS group showed a higher proportion of *Prevotella*, as depicted in Figure 2A. Thus, intrinsic factors related to the host such as aging, disease, or salivary estradiol might condition the oral ecology and subsequently the salivary microbiome.

### 3.4. Oral ecological factors could explain *Prevotella* and *Rothia* modulation in women with Sjögren’s syndrome

**Figure 4:**
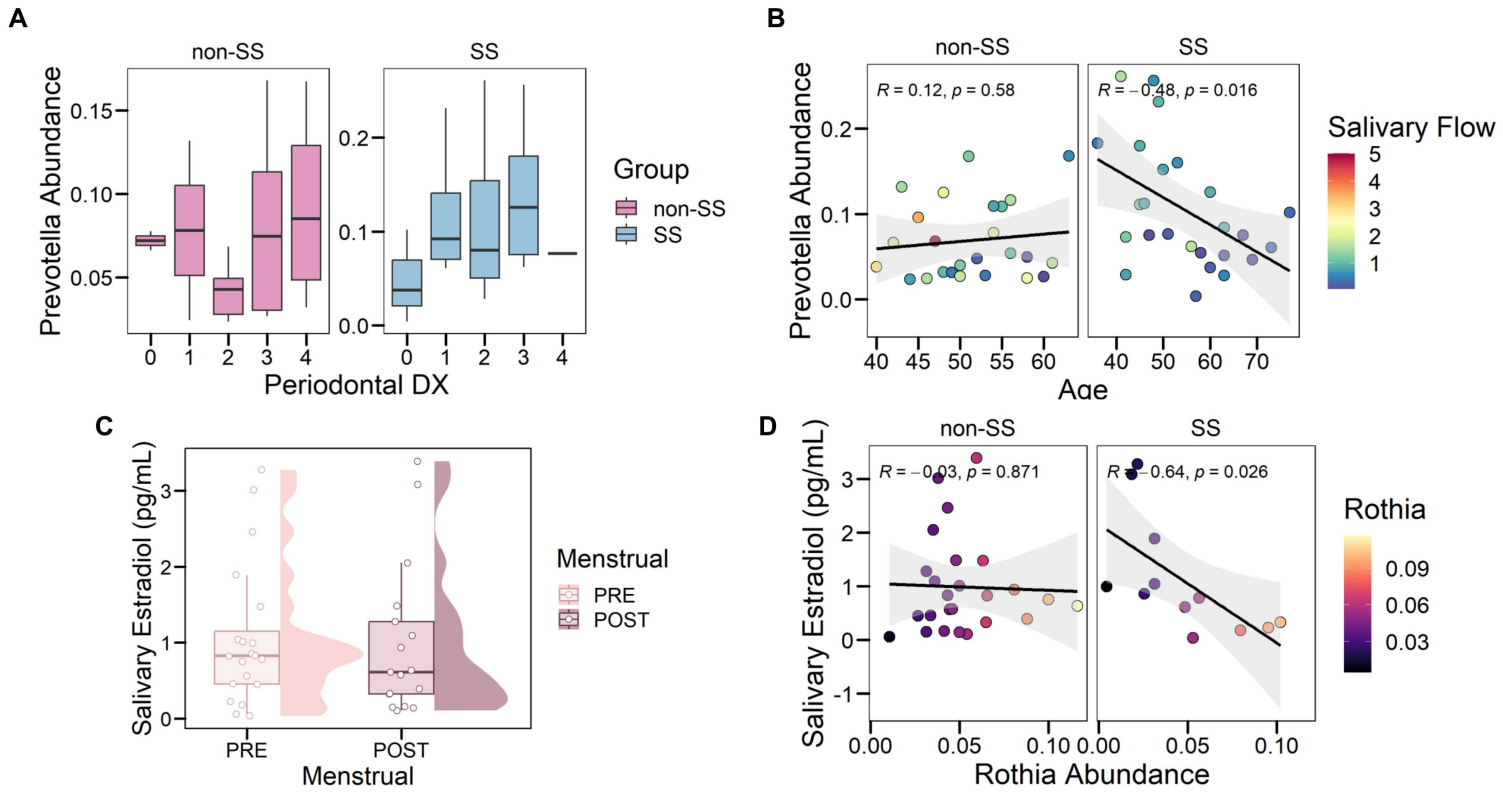
Oral Ecological Factors Modulated *Rothia* and *Prevotella* in Sjögren’s Syndrome. **(A)** Relationship between *Prevotella* abundance and periodontal status. Periodontal categories go from 0 to 4 and read as follows. 0: No treatment required; 1: Instructions for oral hygiene and supragingival prophylaxis; 2: Instructions for oral hygiene, supragingival prophylaxis, localized subgingival prophylaxis and / or removal of overextended fillings; 3: Complete prophylaxis and scaling and root planning of sextants with code 3 (probing depth between 4–6 mm); 4: Supragingival and subgingival prophylaxis, scaling and root planning, and periodontal surgery. **(B)** Association between *Prevotella* abundance and age, dots are colored according to salivary flow (ml/min). **(C)** Boxplot of salivary estradiol levels with respect to menstrual status, including premenopausal women (PRE) and postmenopausal women (POST). **(D)** Correlation between salivary estradiol levels and the relative abundance of *Rothia*.

We investigate whether oral ecological factors explained the variation in microbial age or disease. First, we analyzed the correlation of salivary flow with bacterial abundance. It is important to emphasize that Sjögren’s syndrome might affect the salivary glands. However, this finding strengthens the idea that *Prevotella* was associated with the SS environment even though 92% of the SS patients suffered hyposalivation. It should be noted that *Prevotella* is a known bacterial taxon associated with periodontal disease (Socransky et al., 1998). To investigate the association of *Prevotella* with periodontal disease, we analyzed the periodontal status of women and their relationship with the bacterial taxon (Figure 4A). We found that severe periodontal conditions (codes 3 and 4) were not significantly different from mild periodontal conditions (codes 1 and 2) in women. However, we found that individuals with healthy periodontium had a lower abundance of *Prevotella* compared to periodontal condition only in the SS group, but not significant. Highlight, *Prevotella* decreased with age in the SS group (*R* = −0.48, *p* = 0.016) but varied independently in the non-SS group (Figure 4B, *R* = 0.12, *p* = 0.58).

The oral microbial community can be modulated by intrinsic factors such as salivary estradiol. Therefore, variation in the concentration of the sex hormones might affect the microorganisms harbored in the oral cavity. Since our population was women over 40 years of age and hormones might decrease aging-related, we measured the concentration of salivary estradiol. We classified the menstrual status of women in premenopausal (PRE) and postmenopausal (POST) (Figure 4C). We found that salivary estradiol concentration in women was not significantly different for menstrual status (*p* = 0.66). The genus *Rothia* was negatively correlated with salivary estradiol only in women affected by Sjögren’s syndrome, suggesting that autoimmune disease could affect oral bacteria (Figure 4D, *R* = −0.64, *p* = 0.026). However, *Streptococcus* was not correlated with salivary estradiol for both groups. Overall, we observed that *Prevotella* increased with saliva flow and periodontal status but decreased with age and estradiol levels in women with SS. Interestingly, any of the variables analyzed were not related to non-SS women.

## 4. Discussion

This case–control study aimed to investigate the composition of the oral microbiota in 50 women aged 40 to 70 years, including 25 women with Sjögren’s syndrome and 25 women used as controls. Furthermore, the salivary flow was analyzed to investigate the relationship between hyposalivation and oral microbiota. The results showed a negative correlation between stimulated saliva flow and age, but not between unstimulated saliva flow and age. These findings suggest that saliva production may be affected during stimulation, while no significant changes in salivary flow are observed during rest. In SS, mononuclear cell infiltration and accumulation of adipose tissue cause salivary glands to block (Jonsson et al., 2018). Metabolic abnormalities associated with aging may contribute to this process and have also been linked to the development of SS (Hwang et al., 2021). The implications of the nervous system in SS, including cholinergic dysfunction, may explain the negative correlation between stimulated saliva flow and age in the SS group. Studies suggest independence of salivary gland damage and cholinergic dysfunction, highlighting the role of acetylcholine in salivary gland function in SS (Imrich et al., 2015; Naidu et al., 2022).

In all samples, the most abundant taxa were *Prevotella*, *Neisseria*, *Rothia*, and *Streptococcus*. Consistent with our findings, a similar pattern of abundant taxa was reported in a study by Rusthen et al. (2019) that compared SS and non-SS patients. The oral microbiota can be highly diverse, with several phyla associated with a healthy oral cavity. *Actinobacteria*, *Proteobacteria*, *Firmicutes*, *Bacteroidetes*, and *Fusobacteria* are predominant in the microbiome of healthy individuals (Verma et al., 2018; Li et al., 2022).

Several studies have investigated the association between Sjögren’s syndrome and the oral microbiota. Our study did not find a significant salivary bacterial profile associated with SS disease, contrary to the groups related to salivary production. These results align with those described by Sembler-Møller et al. (2019), who found no significant differences in predominant genera or diversity between 24 SS patients and 36 non-SS controls. Similarly, Sharma et al. reported a comparable trend in their study, identifying three enriched genera such as *Bifidobacterium*, *Lactobacillus*, and *Dialister*, and decreased *Leptotrichia*. However, the study did not find significant differences between the bacterial microbiota of SS and non-SS groups (Sharma et al., 2020). Our results also did not show differences in terms of richness or diversity between the groups, which correlate with previous results (Sharma et al., 2020).

The microbial profile was assessed by separating microorganisms into two dimensions, with *Streptococcus* associated with cluster 1 and *Prevotella* with cluster 2. Interestingly, the non-SS group showed a higher proportion of Streptococcus, while the SS group exhibited a higher abundance of *Prevotella*. Tramice et al. analyzed the oral microbiome and observed a similar pattern, where Streptococcus and *Prevotella* were key factors in data distribution. However, the study did not explore the underlying reasons for this distribution (Tramice et al., 2022).

In our study, we examined the association between *Prevotella* and periodontal status but found no significant relationship. However, we did observe that healthy individuals with good periodontal health had lower levels of *Prevotella* compared to the SS group. A meta-analysis conducted by Yang et al. (2023) reported a positive correlation between *Prevotella* and periodontitis, suggesting also worse periodontal conditions in individuals with SS. However, it is important to note that the meta-analysis reported significant heterogeneity among the included studies.

Our study revealed a significant negative correlation between *Prevotella* spp. and age in the SS group, which was not associated with a reduction in salivary flow. This finding is consistent with previous reports that have suggested a predominance of *Prevotella* spp. in conditions such as poor oral health and advanced age (Yamashita and Takeshita, 2017). In a study by Deshpande et al. (2018), a negative correlation was also observed between *P. melaninogenica* and age in the esophageal microbiota. However, considering this, a similar pattern should have been observed in the non-SS group as well. Other authors have reported negative correlations between age and other taxa at different oral sites, such as *Porphyromonas endodontalis*, *Alloprevotella tannerae*, *Filifactors* alocis, *Treponema* sp., *Lautropia mirabilis* and *Pseudopropionibacterium* sp._HMT_194 (Schwartz et al., 2021). This, along with our findings, suggests that aging may affect the oral microbiota and salivary flow.

We measured salivary estradiol levels in our study to investigate potential correlations with microbiota species. Seven samples were excluded from the analysis due to extreme hyposalivation. This limitation should be considered for future studies, which could involve the measurement of estradiol levels from alternative samples such as blood. This approach could establish correlations between salivary and blood estradiol levels, while also accounting for the hormonal status of the various individuals. Our results showed higher estradiol levels in the SS group compared to the non-SS group, without any significant differences in menstrual status. Previous research has suggested a negative association between estradiol levels and xerostomia (Agha-Hosseini et al., 2009; Soundarya et al., 2022). Soundarya et al. (2022) reported salivary estradiol levels in menstruating women, premenopausal women, and postmenopausal women. In contrast to our findings, their study observed higher mean estradiol levels in premenopausal women than in postmenopausal women. However, women with SS may have lower estrogen levels, as previously reported in the literature (McCoy et al., 2020), which may be related to the role of estradiol in the molecular pathways of salivary glands. These glands contain estrogen receptors (ER), specifically subtypes α and β which are predominantly found in the oral epithelium (Tsinti et al., 2009). ERα promotes proliferation, while ERβ triggers apoptosis through the activation of p38 upon stimulation (Galluzzo et al., 2007). Estradiol, which is detected in salivary glands by these receptors, has a specific role in the physiopathology of Sjogren’s syndrome that has not yet been fully understood. However, previous research has shown a negative correlation between oral dryness and 17-β estradiol concentration, indicating that some aspect of the estrogen receptor pathway may be affected in SS (Agha-Hosseini et al., 2009).

In our study, we observed a negative correlation between salivary estradiol levels and the abundance of *Rothia* in the SS group. Previous research has reported a higher abundance of *Rothia* in breast tumor tissues, and estrogen is known to be a risk factor for this type of tumor (Kartti et al., 2023). However, we did not find any studies that specifically examined the relationship between sex hormones and *Rothia* species. In other species, such as *Streptococcus equi*. Subsp. Zooepidemicus, the presence of the β-glucuronidase gene has been reported (Krahulec and Krahulcová, 2007), suggesting a possible link between the metabolism of sexual hormones and microorganisms.

In conclusion, the oral microbiome is not solely determined by SS but rather by the hyposalivation condition. Stimulated saliva flow showed a negative correlation with age, indicating a decline in saliva production after stimulation, which can be further exacerbated in SS. Two main clusters of microorganisms were identified in the hyposalivation microbiome. *Rothia* and *Prevotella* showed a negative correlation with estradiol and age only in SS women, respectively. These findings contribute to our understanding of the salivary microbiome in the context of hyposalivation, aging, and disease.

## Data availability statement

The datasets presented in this study can be found in online repositories. The names of the repository/repositories and accession number(s) can be found at: https://www.ncbi.nlm.nih.gov/, PRJNA983278.

## Ethics statement

The present study was approved by the Ethics Committee of CEIC Hospital Universitario Ntra. Sra. De Valme 1,532-N^−21^ and European University CIPI/22.201. The research was carried out following the guidelines of the Declaration of Helsinki on Medical Research in Human Subjects and Good Clinical Practice. Written informed consent was obtained from all participants. The studies were conducted in accordance with the local legislation and institutional requirements. The participants provided their written informed consent to participate in this study.

## Author contributions

MJR, MRN, CB, CC-S, MC-O, and DL-C: sample collection and dental examination. MRN, SA-H, LM-G, and JJS-E: recruitment of subjects and acquisition of clinical data. CS, MJR, and AS-S: conception and design of the study, data analysis. CS, MJR, AS-S, and MZ-G: data interpretation and drafting of the manuscript. All authors critically reviewed the manuscript and gave their final approval of the version to be published.

## Abbreviations

SS: Sjögren’s syndrome
SA: unstimulated saliva
ST: stimulated saliva
Normo: normosalivation
Hypo: hyposalivation
PRE: premenopause
POST: postmenopause
ER: estrogen receptor
